# Effect of spectacle correction on hyperopic children

**DOI:** 10.7150/ijms.93822

**Published:** 2024-05-13

**Authors:** Chia-Wei Lin, Cai-Mei Zheng, Yo-Chang Chen, Fu-Gong Lin, Ching-Long Chen, Yun-Hsiang Chang, Jiann-Torng Chen, Jing-Quan Zheng, Ming-Cheng Tai, Yu-Han Huang, Yuh-Feng Lin, Hsin-Ting Lin

**Affiliations:** 1 Department of Urology, Shuang Ho Hospital, Taipei Medical University, Taipei, Taiwan.; 2 Department of Urology, School of Medicine, College of Medicine, Taipei Medical University, Taipei, Taiwan.; 3 Graduate Institute of Clinical Medicine, College of Medicine, Taipei Medical University, Taipei, Taiwan.; 4 Division of Nephrology, Department of Internal Medicine, School of Medicine, College of Medicine, Taipei Medical University, Taipei, Taiwan.; 5 Division of Nephrology, Department of Internal Medicine, Shuang Ho Hospital, Taipei Medical University, New Taipei City, Taiwan.; 6 TMU Research Center of Urology and Kidney, Taipei Medical University, Taipei, Taiwan.; 7 Beauty-Bright Eye Clinics, Zhubei City, Hsinchu County, Taiwan.; 8 Department of Optometry, University of Kang Ning, Taipei, Taiwan.; 9 School of Public Health, National Defense Medical Center, Taipei, Taiwan.; 10 Department of Ophthalmology, Tri-Service General Hospital, National Defense Medical Center, Taipei, Taiwan.; 11 Graduate Institute of Medical Sciences, National Defense Medical Center, Taipei, Taiwan.; 12 Division of Pulmonary Medicine, Department of Internal Medicine, Shuang Ho Hospital, Taipei Medical University, New Taipei City, Taiwan.; 13 Division of Pulmonary Medicine, Department of Internal Medicine, School of Medicine, College of Medicine, Taipei Medical University, Taipei, Taiwan.; 14 Graduate Institute of Aerospace and Undersea Medicine, National Defense Medical Center, Taipei, Taiwan.; 15 Department of Radiology, Tri-Service General Hospital, National Defense Medical Center, Taipei, Taiwan.; 16 Department of Urology, Tri-Service General Hospital, National Defense Medical Center.

**Keywords:** Hyperopia, Spherical equivalent, Visual acuity, Spectacle correction

## Abstract

**Background:** Hyperopia is a significant refractive error in children, often leading to vision impairment. This study aimed to investigate whether partial or full spectacle correction is benefit for hyperopia in preschool-aged children.

**Methods:** A retrospective study was conducted on hyperopic children visited to teaching medical center outpatient clinic between October 2011 and October 2018, and were categorized into three groups: full correction, overcorrection, and undercorrection. The study was approved by the institutional ethical committee of Tri-Service General Hospital.

**Results:** Following a minimum of one-year follow-up period, no statistically significant differences were observed in best-corrected visual acuity (BCVA) among children receiving full, over, or under spectacle correction. Notably, the overcorrection group exhibited a significant reduction in spherical equivalent (SE) compared to both the full and under correction groups, indicating a better SE with spectacle overcorrection.

**Conclusions:** Spectacle overcorrection may offer potential benefits for enhancing SE in preschool children with hyperopia. Nevertheless, further investigation through randomized controlled trials is warranted to establish the validity of this approach and its impact on visual outcomes in this hyperopic pediatric population.

## Introduction

Hyperopia, a refractive error condition characterized by a spherical equivalent (SE) refractive error ≥ +2.00 diopters (D) 1-6, is a significant problem in pre-school children due to its association with blurred vision, amblyopia, and subsequent strabismus 7. Both genetic and environmental influences on hyperopia raise the possibility of ethnic differences in prevalence of hyperopia. Ethnic differences in the prevalence of hyperopia have been observed 8, white children having a 19.3% prevalence, Hispanics having a 12.7% prevalence, and the lowest prevalence among Asians (~7%) in children aged 5 to 17 years. The prevalence of hyperopia was 2.4% to 13.2% among 6-year-old children 1, and 5%-6% higher than myopia prevalence among Asian preschool children 9, 10.

The only treatment for hyperopia is refractive correction using spectacles to reduce the need for accommodative efforts to view near objects 11, 12 with the aim of preventing strabismus 12. However, the optimal amount and threshold of hyperopia correction in various populations remains unclear. There is a dilemma regarding spectacle correction of hyperopia, as it has been suggested that it impairs eye growth and emmetropization 13, potentially leading to future persistent hyperopia 13, 14. Conversely, some researchers have suggested improved visual acuity and reduced effort to accommodation with refractive correction of hyperopia 15, 16. Consequently, different guidelines have been suggested regarding the optimal amount and threshold of hyperopia correction in various populations 14, 17, 18.

Clinically, the extent to which spectacle correction is needed among hyperopic children is still unclear. Ingram *et al.* 19 demonstrated impeded emmetropization in hyperopic children consistently wearing glasses, while others reveal no overall difference noted between partial spectacle correction and those without correction 20. A recent systematic review 21 on the use of spectacle correction versus no spectacles on prevention of strabismus, amblyopia, and emmetropization also reached inconclusive findings. However, clinicians' reluctance to correct hyperopia in children may be unwarranted, as the immediate visual benefit could outweigh their concerns.

Most studies on this topic are limited to very young children with strabismus, and there is little evidence in children of early school years 13. Furthermore, these studies did not fully consider various confounding factors that may affect changes in refractive errors, such as age, initial spherical equivalent (SE), and strabismus 16, 17. Therefore, in this study, we aimed to compare the effects of different spectacle correction (full, under, or over-correction) in early school-age children on the visual acuity (VA) and SE of hyperopic children and to determine if over or under correction could improve spherical equivalent accommodation.

## Methods

### Study cohort

A retrospective study was conducted to assess the impact of different spectacle correction strategies on hyperopic children below the age of 12. We retrospectively analyzed the data from the medical records. The study included 1,185 hyperopic patients who had visited the outpatient clinic between October 2011 and October 2018. Patients with incomplete records, individuals with a spherical equivalent (SE) measurement of less than +1.50 diopters (D) in either eye, and those aged over 12 years at their initial clinic visit were excluded. After applying these criteria, a total of 114 subjects were included in the study. The study was ethically approved on October 24, 2019, by the institutional ethical committee of Tri-Service General Hospital (TSGHIRB 2-108-05-106). Notably, informed consent was not required for this study as it was considered minimal risk and observational in nature. The research adhered to guidelines established by the joint institutional review board and governmental regulations.

### Measurements and patient assignments

The patients with a full cycloplegic spherical equivalent (SE) of at least +1.50 D in both eyes, who were followed up for at least one year, were included in the study. The patients were retrospectively divided into three groups based on their spectacle correction: undercorrection, full correction, and overcorrection. Undercorrection, full correction, and overcorrection were defined as an undercorrection, full correction, and overcorrection of at least 1.00 D of their cycloplegic SE, respectively. Full correction was defined as the difference between the spectacle and cycloplegic SE being less than 1.00 D.

### Statistical analysis

The collected data were analyzed using SPSS for Windows (Ver. 16.0, SPSS Inc., Chicago, IL). The SE data were compared between groups, and multiple comparisons were calculated. The GEE regression model was used to examine the influence of different factors on the magnitude of the absolute change in SE. The results with P <0.05 were considered statistically significant.

## Results

Table 1 presents the baseline characteristics of the study cohort. A total of 114 subjects with hyperopia were included in the study, comprising 60 boys (53%) and 54 girls (47%) with a mean age of 6.24 years (SD 1.62). All subjects were followed for more than 1 year, with an average follow-up time of 41.3 months (mean, SD 19.46). The baseline values for SE, best corrected visual acuity (BCVA), and spectacles at first visit for both the right (OD) and left (OS) eyes were also listed. Additionally, Table 1 presents the final BCVA and changes in BCVA between visits for both eyes. No significant differences were observed among these baseline values.

In Table 2, the initial SE and BCVA values in OD and OS eyes at the first OPD visit were compared among the under full correction, under correction, and overcorrection groups. The majority of the patients were found to be under corrected in both OD (n=90) and OS (n=87) eyes. The SE of both OD and OS spectacles in the undercorrection group was significantly lower than that in the full correction and overcorrection groups (p<0.01). The BCVA of the undercorrection group was significantly worse at the first visit compared with the full correction and overcorrection groups (p<0.01). However, the final BCVA reached almost 1.0 with no significant difference between the three subgroups. The undercorrection group showed a significantly higher change in BCVA in both OD and OS eyes compared with the full correction and overcorrection groups (p<0.01).

Table 3 and 4 present the results of evaluating the improvement in best corrected visual acuity (BCVA) among different spherical equivalent (SE) groups. The study included 114 patients with SE≥2 diopters in both right (OD) and left (OS) eyes (95 and 98 patients, respectively) and 35 patients with SE<2 diopters (19 and 16 patients, respectively). Patients with SE≥2 diopters had a significantly higher improvement in BCVA than those with SE<2 diopters (p<0.01 in OD; p<0.05 in OS). Table 5 shows the changes in SE among the three subgroups of spectacle correction (full correction, undercorrection, and overcorrection). The overcorrection group had a significant decrease in SE after follow-up compared to full and under correction (OD -0.28, OS -0.49, p<0.05 and OD & OS -0.40, p<0.05). Additionally, the average monthly reduction in SE was noted to be -0.02 per month in all patient groups (p<0.01).

## Discussion

Our study evaluated preschool-aged patients (mean age 6.24±1.63 years) and found that most of them were under-corrected for hyperopia. This resulted in significantly worse initial BCVA at the first OPD visit, as shown in Table 2. However, the final BCVA reached nearly 1.0 with no significant difference between the three subgroups (Table 3). Patients with SE≥2 had significantly higher improvements in BCVA compared to those with SE<2, as demonstrated in Table 4. Additionally, a significant improvement in SE was noted among the overcorrection group compared to the full- and under-correction groups, as shown in Table 5. Our study highlights that overcorrection, as compared to full- or under-correction, resulted in similar BCVA with a better improvement in SE.

Our findings suggest that insufficient spectacles may have contributed to the worse initial BCVA observed in the undercorrection group. There are currently no clear recommendations for spectacle correction of hyperopia in the absence of esotropia or reduced visual acuity. The American Optometric Association guidelines for hyperopic children were not clearly recommended 22. A survey of optometrists and pediatric ophthalmologists 13 revealed that only 42% of pediatric ophthalmologists prescribed spectacles for preschool children with >3 D of hyperopia. Our study's results are consistent with these findings, as most of our patients were under-corrected and had a lower initial BCVA at the first OPD visit.

Birch *et al.* 23 identified reduced stereoacuity, anisometropia, and family history of accommodative esotropia as crucial risk factors for the development of accommodative esotropia in hyperopic children. The study conducted by Dobson and Sebris 24 demonstrated that the majority of hyperopic infants had emmetropic vision by the age of three, which may explain the conflicting findings regarding the effectiveness of spectacle correction. However, uncorrected hyperopia in preschool and kindergarten children was associated with significant difficulties in learning to read and write, as demonstrated by lower Test of Preschool Early Literacy (TOPEL) scores 25. Therefore, it is essential to provide spectacle correction to preschool children to avoid any disruption in their language development. Our study found that the BCVA of preschool children with hyperopia improved to approximately 1 after at least one year of follow-up, regardless of whether they were under-, fully-, or overcorrected. The improvement in BCVA was significantly greater among patients with baseline SE≥2 as compared to those with SE<2. This suggests that children with moderate to high hyperopia may benefit more from spectacle correction.

In our study, we observed a significant decrease in spherical equivalent (SE) among the overcorrection group compared to the full and under correction groups. We noted an average monthly reduction in SE of -0.02 in all groups. Previous studies have shown that hyperopia tends to improve with age, which is associated with an increase in axial length and development of myopia 26-29, and believed to be related with the increase in the axial length and development of myopia. However, we found that overcorrection with spectacles was associated with a significant reduction in SE compared to full or undercorrection. This is the first study to demonstrate this effect. One possible mechanism for this effect is that spectacle wearing might have related with a decrease in distance of distinct vision, which improves hyperopic shift. Another possible mechanism might be due to an increase in intra-ocular pressure related with nearer distance 30. Some studies have already proved an increase in axial length associated with increase intraocular pressure 31. There might be a possibility that our patients who tolerated over and full correction in this study had low accommodative amplitudes. Further clinical studies are needed to clarify the accommodative amplitudes and facilities related with spectacle correction among hyperopic children.

Certain limitations exist in our design such as being a retrospective study with no proper randomization, and lack of blinding of patients. Additionally, most of the patients in our study were undercorrected and only a small number of patients received overcorrection, which may reflect current clinical practice in ophthalmology clinics. We did not consider important risk factors such as family history or parental history of refractive errors. In addition, as the duration of correction varies for each case, it was suggested that time variables were included as control variables in the analysis and BCVA/SE was divided by the duration of correction before analysis to present them on a per-unit-time basis (e.g., per month or per year), assuming that the effect of correction varied linearly with time. We also adjusted time variable using Generalized Estimating Equation (GEE) analysis as presented at Table 5. Lastly, due to potential discomfort associated with overcorrection, we were unable to identify non-compliant overcorrection patients.

## Conclusion

In conclusion, our study demonstrates for the first time the importance of spectacle overcorrection in preschool hyperopic children, resulting in a significant reduction in SE with similar BCVA improvement as under- or full correction. Our study may not fully provide such correlation due to its small sample size and design constraints. Further large randomized controlled trials are needed to confirm the significance of spectacle correction in this population.

## Figures and Tables

**Table 1:** Baseline characteristics of study patients

Variables (N=114)	N (%)	Mean, SD	p-value
Gender			
Boys	60 (53%)		<0.01
Girls	54 (47%)		
Age (yrs)		6.24 (1.63)	
Follow-up period (months)		41.43 (19.46)	0.53
Full -1 ~ +1		45.94 (24.94)	
Over >= +1		37.78 (17.73)	
Under <= -1		40.83 (18.56)	
**Initial visit**			
SE (OD)		3.67 (1.876)	0.15
SE (OS)		3.83 (1.90)	
SE of right spectacle		0.98 (1.87)	0.85
SE of left spectacle		1.04 (1.88)	
BCVA (OD)		0.68 (0.25)	1.00
BCVA (OS)		0.68 (0.24)	

OD, right eye; OS, left eye; SE, Spherical equivalent; D, diopter; BCVA, best corrected visual acuity

**Table 2:** SE and BCVA according to spectacle correction at initial visit (N=114)

	Correction (N)	Mean	S.E.	p value
SE (OD)	full -1~1 (16)	3.78	0.45	0.69
	over >=1 (8)	3.13	0.41	
	under <=-1 (90)	3.70	0.20	
SE of spectacle (OD)	full -1~1 (16)	3.80	0.41	<0.01**
	over >=1 (8)	4.66	0.53	
	under <=-1 (90)	0.15	0.07	
SE of right spectacle	full -1~1 (16)	0.23	0.45	<0.01**
	over >=1 (8)	1.53	0.64	
	under <=-1 (90)	-3.55	0.20	
Initial BCVA (OD)	full -1~1 (16)	0.88	0.05	<0.01**
	over >=1 (8)	0.86	0.06	
	under <=-1 (90)	0.63	0.03	
	Correction (N)	Mean	S.E.	p value
SE (OS)	full -1~1(19)	3.90	0.33	0.45
	over >=1(8)	3.00	0.40	
	under <=-1(87)	3.89	0.22	
SE of spectacle (OS)	full -1~1(19)	3.84	0.31	<0.01**
	over >=1(8)	4.58	0.40	
	under <=-1(87)	0.10	0.07	
SE of left spectacle	full -1~1 (16)	-0.06	0.42	<0.01**
	over >=1 (8)	1.58	0.65	
	under <=-1 (90)	-3.79	0.20	
Initial BCVA (OS)	full -1~1(19)	0.85	0.03	<0.01**
	over >=1(8)	0.74	0.10	
	under <=-1(87)	0.64	0.03	

*test with ANOVA test

**Table 3:** Change in BCVA before and after spectacle correction among different spectacle correction groups during follow up period

	Correction (N)	Mean	S.E.	p value
Final BCVA (OD)	full -1~1 (16)	1.01	0.01	0.12
	over >=1 (8)	1.00	0.00	
	under <=-1 (90)	0.97	0.01	
Change in BCVA (OD)	full -1~1 (16)	0.13	0.04	<0.01**
	over >=1 (8)	0.14	0.06	
	under <=-1 (90)	0.34	0.02	
Final BCVA (OS)	full -1~1(19)	1.01	0.01	0.13
	over >=1(8)	0.98	0.03	
	under <=-1(87)	0.96	0.01	
Change in BCVA (OS)	full -1~1(19)	0.16	0.03	<0.01**
	over >=1(8)	0.24	0.09	
	under <=-1(87)	0.33	0.02	

*test with ANOVA test

**Table 4:** Change in BCVA before and after spectacle correction among different SE groups during follow up period

	SE (of eyes) group	Mean	S. D.	p value
Change in BCVA (OD)	<2 (19)	0.13	0.03	<0.01**
	>=2 (95)	0.33	0.02	
Change in BCVA (OS)	<2 (16)	0.19	0.05	<0.05
	>=2 (98)	0.31	0.02	

*test with ANOVA test

**Table 5:** Changes in SE of eyes according to spectacle correction in patients after follow up period

	OD	OS	OD & OS
Variable (N=114)	beta	beta	beta #
Constant	4.12	4.85	4.50
Age	-0.09	-0.12*	-0.11
Gender			
Girls	0.23	0.01	0.12
Boys	Ref	Ref	Ref
Correction			
Under- (<=-1)	0.50*	0.38*	0.43*
Over- (>=1)	-0.28	-0.49*	-0.40*
Full- (>-1~<1)	Ref	Ref	Ref
Follow-up (Month)	-0.02**	-0.02**	-0.02**
Eye	-	-	
Left	-	-	0.16
Right	**-**	**-**	Ref

GEE regression model, * p<0.05; ** p<0.01 # No significance difference detected between right and left eyes
